# Pyrolysis Model Development for a Multilayer Floor Covering

**DOI:** 10.3390/ma8095295

**Published:** 2015-09-14

**Authors:** Mark B. McKinnon, Stanislav I. Stoliarov

**Affiliations:** Department of Fire Protection Engineering, 3106 J.M. Patterson Building, University of Maryland, College Park, MD 20742, USA; E-Mail: mckinn@umd.edu

**Keywords:** material flammability, gasification, fire modeling, composites, engineered materials, carpet, ThermaKin, Controlled Atmosphere Pyrolysis Apparatus

## Abstract

Comprehensive pyrolysis models that are integral to computational fire codes have improved significantly over the past decade as the demand for improved predictive capabilities has increased. High fidelity pyrolysis models may improve the design of engineered materials for better fire response, the design of the built environment, and may be used in forensic investigations of fire events. A major limitation to widespread use of comprehensive pyrolysis models is the large number of parameters required to fully define a material and the lack of effective methodologies for measurement of these parameters, especially for complex materials. The work presented here details a methodology used to characterize the pyrolysis of a low-pile carpet tile, an engineered composite material that is common in commercial and institutional occupancies. The studied material includes three distinct layers of varying composition and physical structure. The methodology utilized a comprehensive pyrolysis model (ThermaKin) to conduct inverse analyses on data collected through several experimental techniques. Each layer of the composite was individually parameterized to identify its contribution to the overall response of the composite. The set of properties measured to define the carpet composite were validated against mass loss rate curves collected at conditions outside the range of calibration conditions to demonstrate the predictive capabilities of the model. The mean error between the predicted curve and the mean experimental mass loss rate curve was calculated as approximately 20% on average for heat fluxes ranging from 30 to 70 kW·m^−2^, which is within the mean experimental uncertainty.

## 1. Introduction

Technological advances in polymer chemistry and composite structure throughout the twentieth century have led to a prevalence of engineered materials, which, in many industries, have completely displaced traditional materials. Engineered materials, including synthetic polymers and composites, can be designed to withstand the stresses associated with physical loadings in the built environment as well as or better than traditional materials. Disadvantages of engineered materials over traditional materials include the inherent flammability of most advanced materials and a lack of knowledge concerning their flammability characteristics. Determining the flammability characteristics of composites is particularly difficult because the parameters that define the energetic and physical interactions between the components and layers are generally not well understood.

There are several standard test methods that are commonly used to characterize the flammability of materials. Quasi-one-dimensional radiant heating tests, including the Cone Calorimeter [1] and the Fire Propagation Apparatus [2], can gather data associated with the fire response of a material. The “Pill Test” [3] and the Flooring Radiant Panel Test [4] are two examples of standard tests specific to the flooring industry. These test methods have been successfully used for decades to assign flammability classifications to the materials used to make consumer products or to collect data from which quantitative flammability characteristics may be inferred. Major criticisms of these and other standard fire tests are the limitations on the scenarios that can be realized in a given apparatus and uncertainties in the physical parameters that define these scenarios. These limitations result in an inability to generalize the results and extrapolate beyond conditions of a given test method.

Computational fire models have significantly improved in recent years such that the most common models are capable of predicting the fire response of entire enclosures. Improved understanding of the fire risk of materials in the built environment over a range of orientations and scales provides practitioners of fire models a basis from which to provide better-informed designs [5]. Several fire models have recently been developed that represent condensed-phase materials that can physically interact with the simulated environment to produce realistic predictions of ignition, the evolution of the burning rate, and flame spread [6,7]. A critical component of predictive fire models is the pyrolysis sub-model that represents gaseous fuel production from the condensed phase given a radiant and/or convective heat flux incident onto the material surface. As fire models have become more frequently relied upon and increasingly complicated, it has become evident that more sophisticated pyrolysis sub-models are required to accurately predict fire behavior.

Essential to accurate predictions with comprehensive pyrolysis models are a set of parameters that correspond to material thermophysical properties as well as decomposition kinetics and thermodynamics for the materials of interest. These parameters have been determined and are publicly available for few common engineered materials because the breadth of parameters required to completely define a material and validate the resulting model requires an extensive experimental and analytical effort. Several researchers have attempted to simplify the process of determining these required parameters through novel experimental apparatuses and procedures [8,9] and numerical optimization schemes used in analysis [10,11].

Recently, our group has developed a pyrolysis model parameterization methodology [12] based on a set of experiments designed to decouple thermal degradation from thermal transport to improve the description of physical phenomena in and provide predictive capabilities for comprehensive pyrolysis models. This methodology was successfully applied to model the pyrolysis of non-charring [13] as well as charring polymers [14]. The objective of the current work is to further advance this methodology and to extend its scope to multilayered composites. The particular target of this investigation is a low-pile carpet tile.

Carpet constructed with synthetic polymers is the most common floor covering material in the built environment although it has poor flammability characteristics compared to other flooring materials. Carpet and area rugs accounted for 50.7% of the U.S. floor covering market in 2013, which amounts to an estimated 985 million m^2^ installed that year [15]. Modern carpet consists of a series of complicated layers made from several different polymers. Low-pile carpet tile, a modular form of the flooring material that is commonly found in commercial and institutional occupancies, particularly in high traffic areas, features at least three distinct layers of polymer mixtures. To the knowledge of the authors, the complicated structure and composition of the carpet that is the subject of this work make this the most complicated material ever to be characterized for a pyrolysis model.

The methodology presented here includes constructing a model for each individual layer to identify its contribution to the pyrolysis dynamics of the composite. A complete understanding of the contribution of each layer to the response of the composite provides the capability to engineer composite materials to meet specific flammability performance metrics. The current study also advances the existing methodology by introducing an additional experimental apparatus, the Microscale Combustion Calorimeter [16], to the parametrization process. This apparatus is employed to determine the heats of combustion of the volatile species produced during pyrolysis. Knowledge of the heats of combustion permits the prediction of heat release rates, which are necessary for the calculation of fire growth and the performance of risk assessments.

## 2. Modeling

The ThermaKin numerical pyrolysis modeling environment was used in this work to conduct inverse analyses on experimental data to indirectly measure thermophysical properties and reaction parameters to describe the thermal degradation of the carpet samples. ThermaKin was also used to generate gasification mass loss rate (*MLR*) and temperature predictions for the sample material to validate the measured properties. ThermaKin solves non-steady energy and mass conservation equations accounting for the physical and chemical processes that occur in the condensed phase during pyrolysis.

The sample material is defined geometrically in ThermaKin as a series of layers with specified thicknesses and chemically as material components defined by specific thermodynamic and physical properties that may be defined with temperature dependence. Thermal degradation reactions are accounted for in the model using Arrhenius reaction rates. ThermaKin has recently been expanded to model flame spread along two-dimensional geometries [17] although only one-dimensional geometries were considered in this work. The one-dimensional governing equations for ThermaKin are provided as Equations (1) to (7).

(1) $$\frac{\partial \xi_{j}}{\partial t}=\overset{N_{r}}{\underset{i=1}{\sum }}\nu_{i}^{j}r_{i}-\frac{\partial J_{j}}{\partial x}+\frac{\partial }{\partial x}\left(\xi_{j}\overset{x}{\underset{0}{\int }}\frac{1}{\rho }\frac{\partial \rho }{\partial t}dx\right)$$

(2) $$\overset{N}{\underset{j=1}{\sum }}\xi_{j}c_{j}\frac{\partial T}{\partial t}=-\overset{N_{r}}{\underset{i=1}{\sum }}h_{i}r_{i}-\frac{\partial q}{\partial x}-\frac{\partial I_{ex}}{\partial x}+\frac{\partial I_{rr}}{\partial x}-\overset{N_{g}}{\underset{g=1}{\sum }}c_{g}J_{g}\frac{\partial T}{\partial x}+c\rho \frac{\partial T}{\partial x}\overset{x}{\underset{0}{\int }}\frac{1}{\rho }\frac{\partial \rho }{\partial t}dx$$

(3) $$r_{i}=A_{i}\exp\left(\frac{-E_{i}}{RT}\right)\xi_{k}\xi_{l}$$

(4) $$J_{g}=-\rho_{g}\lambda \frac{\partial \left(\xi_{g}/\rho_{g}\right)}{\partial x}$$

(5) $$q=-k\frac{\partial T}{\partial x}$$

(6) $$\frac{\partial I_{ex}}{\partial x}=-I_{ex}\overset{N}{\underset{j=1}{\sum }}\kappa_{j}\xi_{j}$$

(7) $$\frac{\partial I_{rr}}{\partial x}=\frac{\epsilon \sigma T^{4}}{I_{ex}^{0}}\frac{\partial I_{ex}}{\partial x}$$

Equation (1) is the statement for the conservation of mass of component *j* in terms of the mass concentration of the component, $\xi_{j}$ (kg m^−3^). The statement for the conservation of mass accounts for consumption or production of component *j* due to reactions, the rate of which is defined in Equation (3), mass transport of gaseous components within the condensed phase which is defined in Equation (4), and mass transport associated with contraction or expansion of the material with respect to a stationary boundary (*x* = 0). Equation (2) is a statement for the conservation of energy of the sample in terms of the material temperature, *T* (K). The statement for the conservation of energy accounts for heat flow due to thermal degradation reactions and phase transitions, heat conduction within the condensed phase, which is defined in Equation (5), absorption of radiant heat from external sources defined in Equation (6), radiant heat loss from the material to the environment defined in Equation (7), convective heat transfer due to gas transport, and energy flow associated with contraction or expansion of the material with respect to a stationary boundary (*x* = 0).

The symbols in Equation (1) through Equation (7) are defined as follows: *t* (s) denotes time and *x* (m) is the Cartesian coordinate. *ρ* (kg m^−3^) denotes density, *c* (J kg^−1^ K^−1^) is the heat capacity, *k* (W m^−1^ K^−1^) is the thermal conductivity, *κ* (m^2^ kg^−1^) is the absorption coefficient, ϵ is the emissivity, and *λ* (m^2^ s^−1^) is the mass transport coefficient. All of these thermophysical properties, with the exception of absorption coefficient and emissivity, may be defined as temperature-dependent. $\nu_{i}^{j}$ is the stoichiometric coefficient for component *j* in reaction *i* which is positive when the component is produced and negative when the component is consumed. *h_i_* (J kg^−1^) is the heat absorbed or released in each reaction or phase transition and may be defined as temperature dependent. *A_i_* ((m^3^ kg^−1^)^n−1^s^−1^) (for reaction of order n) is the Arrhenius pre-exponential factor for reaction *i*, *E_i_* (J mol^−1^) is the activation energy for reaction *i*, and *R* (J mol^−1^ K^−1^) is the universal gas constant. *I_ex_* (W m^−2^) is defined as the radiation flux from external sources traveling within the material, the superscript 0 in $I_{ex}^{0}$ denotes net external radiation flux through the material boundary, and *ϭ* (W m^−2^ K^−4^) is the Stefan-Boltzmann constant.

The thermophysical properties appearing without subscripts correspond to the property of a mixture whereas those with subscripts denote the property of an individual component. The mixture density is calculated as one divided by the sum of the component mass fraction divided by the component density for all components. ThermaKin has the ability to model intumescence by scaling the contribution of gases to the overall volume by a factor related to the local composition. The gases were assumed not to contribute to the volume of the sample in all simulations conducted in this work. The change in thickness due to melting of a fibrous layer of the carpet was simulated by a prescribed increase in the density associated with this transition.

The ThermaKin program divides the computational domain into rectangular control volumes (elements) and calculates the temperature and mass of each component in all the elements for each time step. The rate of change of each property is defined as a function of the component masses and element temperature. The resulting coupled algebraic equations are linearized and solved at each time step. A detailed description of the physics represented in the ThermaKin modeling environment and the numerical solution approach are provided elsewhere [18].

## 3. Experiments and Analysis

### 3.1. Materials

**Figure 1:**
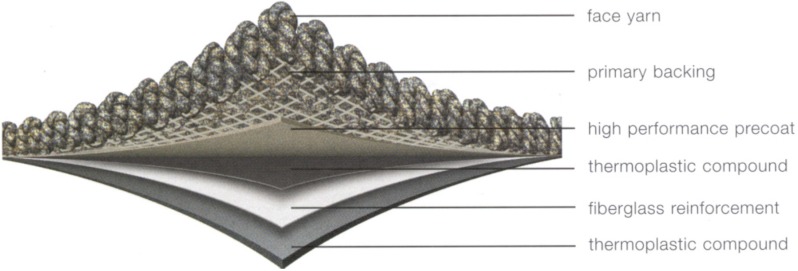
Schematic of the EcoWorx carpet tile [19].

EcoWorx style low-pile carpet tiles produced by the Shaw Contract Group were characterized in this work according to the methodology presented in the following sections. A schematic of a carpet tile sample is provided in Figure 1. The approximate mass of each polymer contributing the largest mass to each layer of the carpet was provided by the manufacturer.

The face yarn is made of 0.42–0.57 kg·m^−2^ of woven polyamide-6 (PA6) with auxiliary polymers. The primary backing, which is composed of a mesh through which the face yarn is interwoven, includes approximately 0.11 kg·m^−2^ of a PA6 and polyethylene terephthalate (PET) bicomponent mixture. The precoat is made from approximately 0.42 kg·m^−2^ of highly-filled vinyl-acetate ethylene (VAE) with other auxiliaries. The base layer consists of approximately 1.18 kg·m^−2^ of highly filled very-low-density polyethylene (LDPE) (labeled as “thermoplastic compound” in Figure 1) with auxiliary additives as well as 0.05 kg·m^−2^ of nonwoven fiberglass mat.

**Figure 2:**
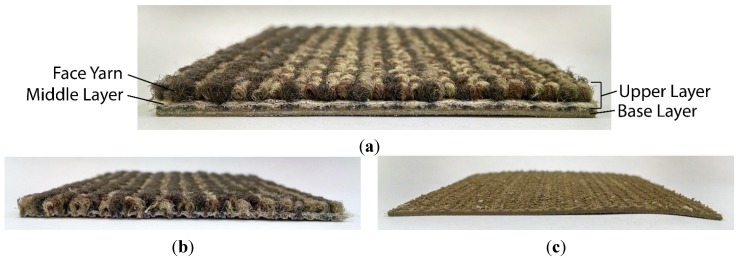
Photographs of (**a**) full carpet composite; (**b**) upper layer; and (**c**) base layer.

The tile was separated by pulling the composite into two layers denoted as the upper layer and the base layer. Coupon-sized samples of the two individual layers were tested in bench-scale experiments to characterize thermal transport through the composite. The two layers tested in bench-scale experiments are displayed in Figure 2b,c together with a photograph of the full carpet sample in Figure 2a.

Samples of each of the three individual layers sized on the order of several milligrams were harvested and tested to characterize the thermal degradation reaction kinetics, energetics, and combustibility of the gases evolved during thermal degradation. These three layers were the face yarn, the middle layer, which consisted of the primary backing and the precoat, and the fiberglass-reinforced base layer. Because of their small size, these samples were easily cut from the upper layer and base layer while keeping the structure of each layer intact.

The masses and thicknesses of coupon-sized samples (0.08 m × 0.08 m) of the entire carpet, the upper layer, and the base layer were measured to verify the geometric and gravimetric definitions of the individual layers in the successive analyses described in Section 3.2.4 and to confirm the additive nature of each layer to the composite. The thickness of the face yarn in the upper layer sample was measured, all the face yarn was carefully removed from the sample, and the mass and thickness of the remaining middle layer portion was measured to determine the relative masses of the face yarn and middle layer in the upper layer. The areal density of the face yarn was calculated as the compliment to the areal density of the middle layer in the upper layer samples.

The areal density of the base layer was measured as 1.770 ± 0.060 kg·m^−2^ and the thickness of the layer was measured as 0.0017 ± 0.0001 m. The areal density of the face yarn layer was measured as 0.350 ± 0.050 kg·m^−2^ and the thickness of the layer was 0.0030 ± 0.0002 m. The areal density of the middle layer was measured as 0.970 ± 0.050 kg·m^−2^ and the thickness was measured as 0.0016 ± 0.0003 m. These measurements led to the following definitions for the density of each virgin component: The ${\text{Face Yarn}}_{\text{virgin}}$ component density was defined as 125 kg·m^−3^, the ${\text{Middle}}_{\text{virgin}}$ component density was defined as 582 kg·m^−3^, and the ${\text{Base}}_{\text{virgin}}$ component density was defined as 1060 kg·m^−3^ in individual layer models constructed as described in Section 3.2.4.

The areal densities of the individual layers were additive to within the uncertainty of the measured areal density of the composite, but the thicknesses did not add to within the uncertainty of the thickness of the composite. The disparity between these two measurements originated from a textured interface between the base layer and the precoat component in the middle layer (see Figure 2). The thickness of the middle layer was measured as approximately 0.0011 ± 0.0001 m as a layer in the composite and the thickness of the base layer was measured as 0.0015 ± 0.0001 m as a layer in the composite.

The densities of the base layer and middle layer components defined in the individual layer models were modified during construction of the full carpet composite model to maintain consistency with the measured masses and to account for the reduced thicknesses of these layers in the tested composite. The density definitions for the middle layer and base layer were modified to the following values: the ${\text{Middle}}_{\text{virgin}}$ component density was defined as 750 kg·m^−3^, and the ${\text{Base}}_{\text{virgin}}$ component density was defined as 1200 kg·m^−3^ in the full carpet composite models. Construction of the model that required these geometric and gravimetric definitions is outlined in Section 3.2.4.

### 3.2. Experimental Methods

#### 3.2.1. Simultaneous Thermal Analysis

Simultaneous Thermal Analysis (STA) is a generic term for a class of thermal analysis methods that are simultaneously conducted with a single apparatus. In this work, Thermogravimetric Analysis (TGA) and Differential Scanning Calorimetry (DSC) tests were conducted together to measure the sample mass and heat flow rate to the sample as a function of sample temperature and time. The apparatus used in this work was a Netzsch 449 F3 Jupiter STA (Netzsch Group, Selb, Germany). The apparatus constantly measures the temperature and mass of sample and reference crucibles located next to each other in a test chamber while the chamber temperature is scanned through a well-defined program. The heat flow rate to the sample is correlated to the temperature difference between the reference and sample crucible through a calibration curve that relates this temperature difference to the known heat of fusion of anhydrous salts that melt throughout a wide temperature range. A temperature calibration was also conducted in which the melting point of the anhydrous salts was related to the measured temperature at the phase transition. Details of these calibration procedures are provided elsewhere [20]. Both the heat flow and temperature calibrations were verified periodically throughout the course of the current study. The calibrations were conducted using the same exact test conditions and crucibles as those utilized for the carpet sample analysis.

The temperature program for the STA tests was designed with an initial conditioning period to allow the sample to equilibrate with the atmosphere at a constant temperature of 313 K for 20 min and to ensure the sample was purged of oxygen and residual moisture before dynamic data was collected. The conditioning period was followed by linear heating at a rate of 10 K·min^−1^ to approximately 100 K above the highest temperature at which mass loss was observed. This heating rate was chosen to ensure the samples did not experience significant temperature or mass gradients, which created conditions where the thermal degradation kinetics and energetics were decoupled from heat and mass transport within the sample. The test chamber was constantly purged with nitrogen flowing at a rate of 50 mL·min^−1^ to investigate thermal degradation while eliminating oxidation and other unwanted heterogeneous reactions.

Ten STA tests were conducted on samples of each of the three layers to accumulate the necessary statistics. The sample material was cut to a size such that the total mass of the sample ranged from 3 to 10 mg. The sample was compacted flat against the bottom of a platinum crucible. Thermal contact between the sample and the crucible proved to be an important variable and it was observed that reliably repeatable data was obtained when the sample was positioned in the crucible consistently between tests. All STA tests were conducted with the crucible lid covering the sample to ensure a uniform temperature profile within the sample. An opening in the center of the lid allowed pyrolyzate to escape the crucible.

Analysis of STA data required a model constructed in the ThermaKin modeling environment that simulated the STA tests. In this model, the convection coefficient at the sample boundaries was defined sufficiently high to force the boundaries of the sample to adhere to the ambient temperature. The mass flow boundary conditions were defined such that the gaseous pyrolyzate instantaneously escaped the sample. The sample thickness was defined small enough to guarantee uniformity of the sample temperature and component concentrations throughout the sample. In previous studies where similar modeling was carried out [20,21], the time evolution of the ambient temperature was prescribed in accordance with the experimental heating rate set point. In the current study, the heating rate was defined to follow its approximate measured temporal evolution, which significantly improved the modeling of the early test stages, where the sample heating rate fluctuated significantly prior to reaching its set point. The model was used to calculate the sample mass loss and heat flow throughout thermal degradation.

The mass and *MLR* data collected in STA tests were analyzed through a manually iterative inverse analysis procedure to determine the thermal degradation kinetic mechanism. The initial number of reactions was determined through a visual inspection of the TGA and DSC data, and all reactions were assumed to be of first order (*i.e.*, one of the component concentrations in Equation (3) remained fixed at unity). While analyzing the mass data, an attempt was made to use the fewest reactions possible to accurately describe the experimental curves to reduce unneeded complexity in the model. Additional reactions were only included into the mechanism when doing so improved the agreement between the model predicted curves and the experimental curves to within the acceptance criteria described later in this section. It is important to note that the reaction mechanism and the components included in the mechanism do not represent actual elementary reactions or individual chemical components. The mechanism is intended to mathematically mimic the mass and *MLR* data in the simplest form possible and relies on model-specific kinetics to reproduce these data.

In each case, the first reaction curve that was fit corresponded to the largest *MLR* peak. The temperature at the peak *MLR* (*T*_peak_), the peak *MLR* (*MLR*_peak_), the initial reactant mass (*m*_0_) and the condensed-phase product yield (*v*) were required to determine the Arrhenius reaction parameters (*A* and *E*). An initial estimate of the activation energy was calculated from Equation (8), which is an approximate solution to the first order Arrhenius kinetics under linear heating conditions [22].

(8) $$E=\frac{eRT_{\text{peak}}^{2}{\mathrm{MLR}}_{\text{peak}}}{\left(1-\nu \right)m_{0}\frac{dT}{dt}}$$

The pre-exponential factor corresponding to the initial estimate of the activation energy was calculated using Equation (9).

(9) $$A=\frac{e{\mathrm{MLR}}_{\text{peak}}}{(1-\nu )m_{0}}e^{\frac{E}{RT_{\text{peak}}}}$$

After the initial estimate, the agreement between specific traits of the experimental curves and the curves predicted through the ThermaKin simulation were evaluated and the parameters were recalculated if an improved agreement was required. General guidelines were followed to adjust the reaction parameters during the curve fitting process. By increasing the activation energy, the *MLR* curve shifted to higher temperatures and by increasing the pre-exponential factor, the magnitude of the peak increased. The activation energy was iteratively modified and the pre-exponential factor was recalculated until acceptable agreement was achieved. The integral of the *MLR* curve is associated with the total mass lost during the thermal degradation process, and it was influenced by adjusting the stoichiometry of the reaction. If acceptable agreement could not be obtained using the reactions already defined in the model, one additional reaction was added. Acceptable agreement was defined as a maximum error of 10% in the prediction of the magnitude of *MLR*_peak_, a maximum absolute error in the prediction of $T_{\text{peak}}$ of 3 K, and a mean error of less than 2% in the normalized mass versus temperature curve. The resulting reaction schemes are presented in Section 4.1.1.

The reaction mechanism defines all of the components present in the sample, the rates of transition of those components to solid degradation products, and the evolution of the mass of all components with respect to time and temperature. The solid products of degradation may be the final products, usually termed char, or may be intermediate products that eventually degrade to the final products. This understanding of the changes in the composition of the solid sample as a function of temperature provides the foundation for the analysis of heat flow rate data from STA tests.

The parameters required to completely account for heat flow to the sample include the heat capacity of each component (excluding gases because they are assumed to leave the crucible instantaneously), the heat absorbed in each reaction and phase transition, and the reaction and phase transition rates with respect to temperature. The heat capacity was calculated by dividing the heat flow rate measured in STA tests by the observed heating rate. The range of data analyzed corresponded to the initial, unreacted components. The heat capacity of the char components was generally assumed or determined from STA tests conducted on the char samples. The intermediate components that were produced and consumed in reactions during degradation were assumed to have the mean heat capacities of the component that reacted to produce the intermediate and the component that was produced when the intermediate was consumed.

A baseline heat flow rate curve that accounts for the sensible enthalpy as a function of temperature throughout degradation was constructed using the component mass fractions predicted by the kinetic mechanism and the determined heat capacity values. The remaining contributions to the heat flow rate were due to reactions and phase transitions. The heats absorbed by the sample specimen during these chemical reactions and phase transitions were determined as the integral between the experimental heat flow rate curve and the sensible enthalpy baseline over the temperature range that corresponded to the reaction or phase change. Because the DSC data was normalized by the initial sample mass, the integral values were corrected to the total available mass of the corresponding reactant.

The preceding analysis yielded all the parameters required to predict the heat flow rate to the sample as a function of temperature. The STA experiment was simulated and the predicted heat flow rate curve was compared to the mean experimental curve to ensure that all heat flow events were adequately described by the reaction mechanism. Modifications were made to the heat capacities or the reaction mechanism if the comparison indicated that the predictions yielded from this analysis were unacceptable. The acceptance criterion for this analysis required that the relative difference between the time-dependent integrals of the predicted heat flow rate curve and the experimental curve remain less than 5% to verify the total energy absorbed by the sample in the model was equivalent to the total energy absorbed by the sample in the experiment throughout decomposition.

#### 3.2.2. Microscale Combustion Calorimetry

Microscale Combustion Calorimetry (MCC) is a testing method in which a sample is pyrolyzed in an inert atmosphere as the sample is heated according to a well-defined temperature program [23]. The gases evolved during pyrolysis flow to a combustion chamber where combustion occurs with excess oxygen and the heat release rate from the combustion reaction is measured using oxygen-consumption calorimetry.

The heats of combustion of the gases produced during thermal degradation of carpet samples were determined through an analysis of MCC data in which the heat release rate (*HRR*) temperature dependencies were compared to the corresponding *MLR* curves predicted by the kinetic mechanism determined through analysis of STA data. The heats of combustion relate the rate of production of gaseous pyrolyzate to the *HRR* of the sample at a range of geometric scales. MCC data were also used to verify the reaction mechanism determined through analysis of STA data. The MCC data made it possible to determine whether the complexity of the reaction mechanism determined through analysis of STA data was sufficient to represent the changes in combustibility of gaseous products evolved throughout degradation.

An apparatus built in accordance with the standard [16] was used to conduct MCC tests. The oxygen sensor was calibrated against a standard O_2_/N_2_ mixture to ensure accurate *HRR* measurements. A temperature calibration that relied on the melting point of several pure metals was conducted to ensure accurate sample temperature measurements [24]. The combustor temperature was maintained at 900 K.

Three MCC tests were carried out on samples of each of the face yarn, middle layer, and base layer. Samples for MCC tests were prepared identically to the samples tested in the STA. The sample mass, which ranged from approximately 2 to 5 mg was recorded and the sample was placed in a ceramic crucible. The tests were conducted without lids on the crucibles to allow all pyrolyzate gases to escape unimpeded. The crucible was introduced to the pyrolysis chamber and allowed to reach equilibrium at 348 K. Upon reaching equilibrium, the temperature of the pyrolysis chamber was increased linearly at a heating rate of 10 K·min^−1^ to the final temperature of approximately 1023 K. The heating rate used in this investigation is outside the range of heating rates recommended in the standard, but was chosen to provide data collected under conditions comparable to those used in the STA tests.

Though the set point heating rates were identical between MCC and STA tests, the observed heating rate profiles included transients that were considerably different for each apparatus. Because of these differences, a direct comparison between the experimental data from each apparatus could not be made. A comparison between the data required the prediction of a *MLR* curve using the reaction mechanism determined through analysis of the STA data simulated at the heating rate profile observed in the MCC experiment. The measured *HRR* curve was divided by the predicted *MLR* curve for each distinct pyrolyzate species to yield a heat of complete combustion value for the gaseous pyrolyzate evolved in each reaction.

A predicted *HRR* curve was generated by simulating the mass loss process in the MCC experiment and applying the heat of combustion value to each distinct gaseous species. The predicted *HRR* curve was compared to the experimental *HRR* curve. Modifications were made to the heat of combustion values until the predicted and experimental curves agreed to within the acceptance criterion. Though qualitative agreement between the experimental and modeled *HRR* curves was important for determining the heat of combustion for each modeled gaseous species, the only formal acceptance criterion required that the total integrals of the predicted curve and the experimental curve agree to within 2%.

#### 3.2.3. Absorption Coefficient Measurement

**Figure 3:**
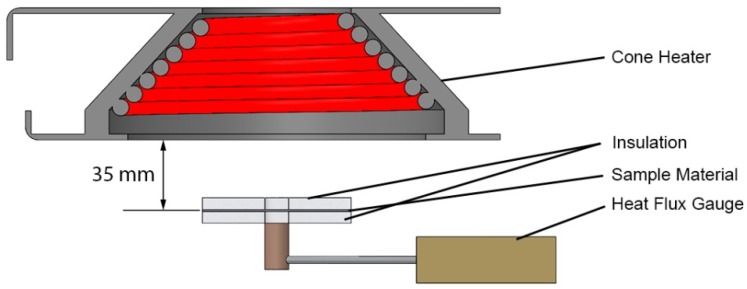
Rendering of the method used to measure absorption coefficient.

A method loosely based on a technique described by Linteris, *et al.* [25] was used to estimate the absorption coefficient of each of the layers in the carpet composite. The method involved irradiating a sample with a thickness on the order of 0.001 m at a heat flux of approximately 35 kW·m^−2^ (980 K) while measuring the radiation flux transmitted through the sample with a Schmidt-Boelter heat flux gauge. A rendering of the measurement setup is provided in Figure 3.

The data collected in this measurement was used to calculate the effective absorption coefficient with Equation (10) adapted from [26]. In Equation (10), *κ* is the absorption coefficient, *γ* is the reflection loss coefficient, *I* is the measured radiant heat flux incident to the surface (*x* = *δ*) and transmitted through the material (*x* = 0), and the sample thickness is denoted as δ. The transmitted heat flux was measured the instant irradiation of the sample began to ensure the conduction through the sample did not contribute significantly to this measurement. The mean steady radiation flux to the heat flux gauge after the sample material was removed was measured and was used as *I_x_*_=_ *_δ_* in Equation (10). The values of the reflection loss coefficient utilized in these calculations are discussed in Section 4.1.3.

(10) $$\kappa =\frac{\left[2\ln(1-\gamma )-\ln\left(\frac{I_{x=0}}{I_{x=\delta }}\right)\right]}{\rho \delta }$$

#### 3.2.4. Gasification Experiments and Analysis

An apparatus that augments the standard Cone Calorimeter [1], the Controlled Atmosphere Pyrolysis Apparatus (CAPA) [9], was designed to generate well-defined conditions in the vicinity of the sample surface while sample mass and back surface temperature (*T*_back_) data are simultaneously collected. The radiant heat flux onto the front surface of the sample is generated by the cone calorimeter heater. The mass of the sample is measured throughout the tests by the mass balance internal to the cone calorimeter system. The apparatus features an infrared camera focused on a gold mirror to reflect the image of the back surface of the sample. The infrared camera records a spatially-resolved temperature measurement of the back surface. The CAPA was designed to enable simultaneous measurement of the sample *T*_back_ and the sample mass throughout the duration of the test.

**Figure 4:**
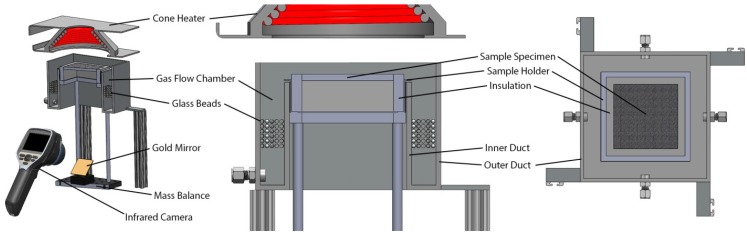
Rendering of the Controlled Atmosphere Pyrolysis Apparatus (CAPA).

The basic design of the CAPA is displayed in Figure 4. The design consists of two concentric, square ducts (inner and outer duct in Figure 4) with a sample holder inside the inner duct. The annular space between the inner and outer duct comprises the gas flow chamber. The gas flow chamber features a flow path through a low pressure drop area followed by approximately 1 inch of glass beads to homogenize the gas flow that is injected in to the gas flow chamber. This design was implemented to uniformly distribute the purge gas along the edges of and over the surface of the sample to maintain a well-defined gas composition above the sample surface.

The interior volumes of the inner duct and sample holder have square cross sections with sides measured 0.120 m and 0.115 m, respectively. The gap between these two is blocked by a lip installed on the sample holder to limit oxygen leakage to the sample surface. The lip is located 0.001 m above the upper edge of the inner duct, ensuring that the sample holder is suspended on the balance and does not make contact with the CAPA ducts to prevent interference with the mass measurement.

The tests conducted in this work with the CAPA had nitrogen introduced to the gas flow chamber at a rate of 225 L·min^−1^ (measured at 1 atm and 298 K). At this flow rate, the mean oxygen concentration approximately 0.001 m from the front surface of the sample was measured as 2.2 ± 0.4 vol %. This oxygen concentration prevented autoignition for all samples and appeared to make any effects of oxidation on *MLR* and *T*_back_ measurements inconsequential.

A Schmidt-Boelter heat flux gauge was positioned at a location corresponding to the geometric center of the front surface of the sample, 0.040 m below the bottom of the cone heater, to set the radiant heat flux to the sample. A feedback control system internal to the cone calorimeter automatically adjusted the temperature of the heater based on the signal from the heat flux gauge. The uniformity of the heat flux at the sample surface was examined with a separate heat flux gauge and was found to be satisfactory (<7% maximum deviation from the set point).

The CAPA tests were conducted on samples of the upper layer, the base layer, and the full carpet composite subjected to radiant heat flux of 30, 50, and 70 kW·m^−2^. Each test was repeated three times to accumulate statistics. Samples were prepared in a square geometry with a side of 0.08 m. They were located in the center of a square sheet of 0.00625 m thick Kaowool PM board with an edge dimension of 0.105 m.

**Figure 5:**
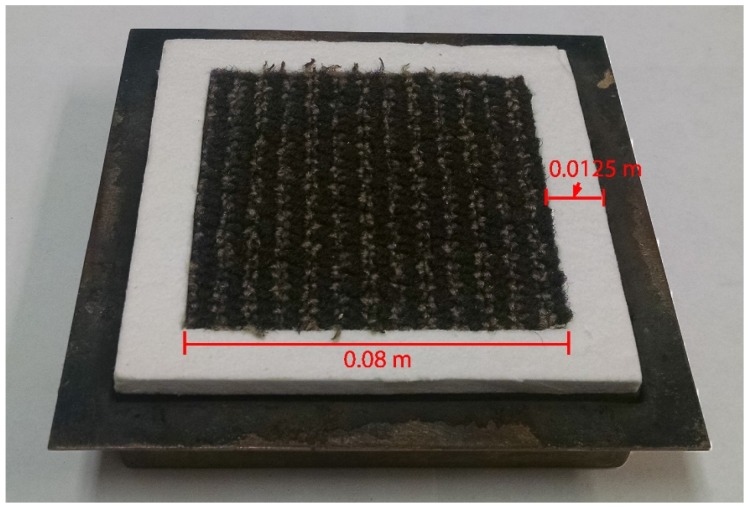
Photograph of a carpet composite sample mounted on the sample holder for CAPA tests.

In the tests on the face yarn and the full composite, the sample rested on a square piece of aluminum foil that was painted on the side facing away from the sample for an emissivity of 0.95 to provide a well-defined surface emissivity for the infrared camera. The base layer samples were tested without aluminum foil and their back surfaces were painted for an emissivity of 0.95. The paint partially degraded above approximately 600 K, compromising the well-defined emissivity, and data collected above this threshold was considered unreliable and was not used in inverse analyses. It was observed during preliminary CAPA tests that the edges of the face yarn samples curled toward the center of the sample immediately upon heating and decreased the exposed area of the sample. The face yarn samples prepared for subsequent CAPA tests were secured to the holder with wires to combat deformation of the samples. A photograph of the sample holder is provided in Figure 5.

Inverse analyses of the data were conducted with the ThermaKin modeling environment to determine the parameters that define thermal transport at the surface and within the condensed phase. Analysis with ThermaKin required a one-dimensional assumption and the spatially-resolved temperature data was reduced to a single representative value of the *T*_back_. The *T*_back_ data was recorded at a rate of 7.5 Hz. In each frame, the image was divided into three regions. Region 1 consisted of the central 0.04 m × 0.04 m square, region 2 consisted of the central 0.06 m × 0.06 m square less region 1, and region 3 consisted of the entire 0.08 m × 0.08 m sample surface less regions 1 and 2. The mean *T*_back_ was calculated in each frame using four randomly selected locations from regions 1 and 2, and two randomly selected locations from region 3. The *T*_back_ was generally uniform, with the maximum deviation from the mean value on the order of 5%.

The inverse analyses consisted of defining an initial value of the thermal conductivity for the desired components and iteratively modifying the value to improve the agreement between the model prediction and the target data. The conditions defined in the model that could not be directly measured were the convective heat fluxes (representing losses) at the front and back boundaries. These heat fluxes were characterized through inverse analyses on data collected in CAPA tests to measure the temperature of a sample-sized copper plate painted for a well-defined emissivity [13].

The boundary condition for the front surface was defined with a radiant heat flux identical to the heat flux set point for the CAPA tests. The convective heat transfer coefficient was defined as 5 W·m^−2^·K^−1^ and the ambient temperature at the front surface was measured as 330 K at 30 kW·m^−2^ and exhibited a linear dependence on the set heat flux up to 370 K at a heat flux of 70 kW·m^−2^. The mass transport at the boundary was defined to provide no impedance to the escape of gaseous pyrolyzate produced during degradation.

The back surface was defined to be impenetrable to mass transport. The convective boundary condition at the back surface of the sample was defined with a heat transfer coefficient of 4 W·m^−2^·K^−1^ and an ambient temperature of 310 K. A radiant heat flux of 500 W·m^−2^ was applied to the back surface to simulate radiation from the internal walls of the test apparatus (which were assumed to be at ambient temperature). The absorption coefficient of the back surface was defined such that all incident radiation was absorbed at the surface. The emissivity of the back surface was defined as 0.95 to simulate the presence of high emissivity paint on the back surface of the tested samples or on the aluminum foil on which the samples rested. A default value for the mass transport coefficient was defined for all components (2 × 10^−5^ m^2^ s^−1^). This value was determined as high enough to allow all gaseous pyrolyzate to escape the condensed phase with no impedance to flow, and low enough that it would maintain the stability of the integration [27].

## 4. Results

### 4.1. Data Analysis for Property Evaluation

#### 4.1.1. Thermal Degradation Kinetics and Energetics Determination

**Figure 6:**
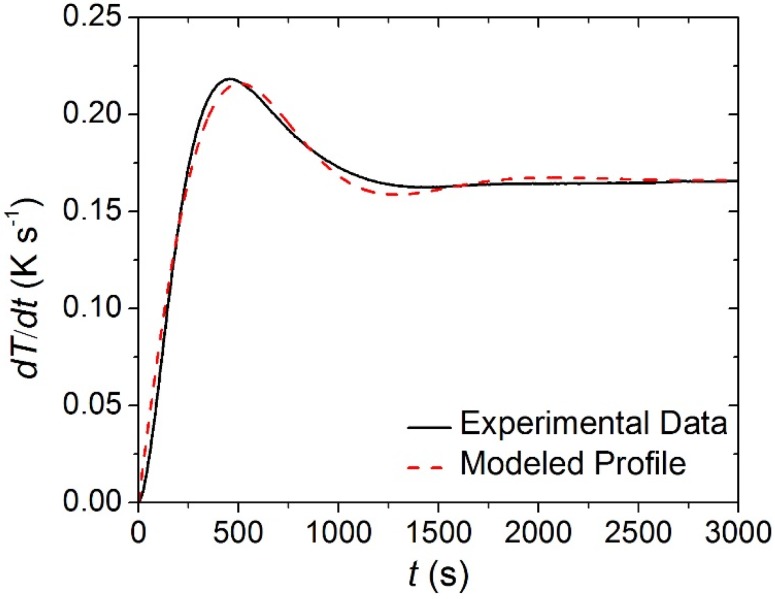
Experimentally observed and modeled heating rate histories typical of the Simultaneous Thermal Analysis (STA). The coefficients for Equation (11) that describe the modeled curve are the following: *a* = 0.166 K s^−1^, *b* = 0.0024 s^−1^, *f* = 0.004 s^−1^, *g* = −0.0623.

The STA data collected on each of the individual carpet layers was analyzed to determine the kinetics and energetics of the thermal degradation process as well as the heat capacity of the condensed phase components. Though the STA followed a well-defined temperature program, the set point heating rate (0.167 K·s^−1^) was not achieved instantaneously. Rather, the heating rate measured in each of the tests had reproducible time-dependency that was approximated in ThermaKin by an exponentially-decaying sinusoid, Equation (11). The parameters of this equation were adjusted until it matched the experimental data. The agreement between the experimentally observed and modeled heating rate profiles is evident in Figure 6.

(11) $$\frac{dT}{dt}\left(t\right)=a\left(1-\left(\exp\left(-bt\right)\right)\left(\cos ft+g\sin ft\right)\right)$$

It was assumed that a mechanism with consecutive reactions would be suitable for the face yarn and the base layer. Analysis of the face yarn layer resulted in a mechanism with one phase transition reaction and two thermal degradation reactions. The mechanism determined for the base layer also featured a single phase transition and two thermal degradation reactions. A parallel scheme was assumed for the middle layer because it was known prior to the analysis that the middle layer was composed of at least four distinct polymers that degraded independently. The pyrolysis mechanism for the middle layer included two parallel phase transitions and four parallel thermal degradation reactions. The effective reaction mechanism determined for each layer is provided in Table 1.

**Table 1:** Effective reaction mechanisms for each layer of the carpet composite and the heats of reactions. Positive heats represent endothermic processes.

#	Reaction Equation	*A* (s^−1^)	*E* (J·mol^−1^)	*h* (J·kg^−1^)
**Face Yarn**				
1	${\text{Face Yarn}}_{\text{virgin}}\to {\text{Face Yarn}}_{\text{melt}}$	6.0 × 10^38^	3.80 × 10^5^	6.1 × 10^4^
2	${\text{Face Yarn}}_{\text{melt}}\to 0.92{\text{Face Yarn}}_{\text{int}.}+0.08{\text{Face Yarn}}_{\text{volatiles}}$	1.0 × 10^9^	1.41 × 10^5^	5.3 × 10^4^
3	${\text{Face Yarn}}_{\text{int}.}\to 0.06{\text{Face Yarn}}_{\text{char}}+0.94{\text{Face Yarn}}_{\text{volatiles}}$	3.0 × 10^14^	2.30 × 10^5^	5.3 × 10^5^
**Middle Layer**				
1	${\text{Middle}}_{4,\text{ virgin}}\to {\text{Middle}}_{4,\text{ melt}}$	9.0 × 10^38^	3.84 × 10^5^	8.0 × 10^4^
2	${\text{Middle}}_{3,\text{ virgin}}\to {\text{Middle}}_{3,\text{ melt}}$	1.0 × 10^28^	3.00 × 10^5^	6.0 × 10^4^
3	${\text{Middle}}_{1,\text{virgin}}\to 0.334{\text{Middle}}_{\text{char}}+0.666{\text{Middle}}_{\text{volatiles}}$	1.0 × 10^12^	1.55 × 10^5^	2.7 × 10^6^
4	${\text{Middle}}_{2,\text{virgin}}\to 0.334{\text{Middle}}_{\text{char}}+0.666{\text{Middle}}_{\text{volatiles}}$	1.0 × 10^20^	2.62 × 10^5^	0
5	${\text{Middle}}_{3,\text{melt}}\to 0.334{\text{Middle}}_{\text{char}}+0.666{\text{Middle}}_{\text{volatiles}}$	5.0 × 10^8^	1.42 × 10^5^	3.5 × 10^5^
6	${\text{Middle}}_{4,\text{melt}}\to 0.334{\text{Middle}}_{\text{char}}+0.666{\text{Middle}}_{\text{volatiles}}$	1.0 × 10^10^	1.70 × 10^5^	2.0 × 10^5^
**Base Layer**				
1	${\text{Base}}_{\text{virgin}}\to {\text{Base}}_{\text{melt}}$	1.0 × 10^21^	1.72 × 10^5^	6.0 × 10^3^
2	${\text{Base}}_{\text{melt}}\to 0.92{\text{Base}}_{\text{int}.}+0.08{\text{Base}}_{\text{volatiles}}$	5.0 × 10^6^	1.15 × 10^5^	4.0 × 10^4^
3	${\text{Base}}_{\text{int}.}\to 0.71{\text{Base}}_{\text{char}}+0.29{\text{Base}}_{\text{volatiles}}$	1.0 × 10^16^	2.58 × 10^5^	1.5 × 10^5^

**Figure 7:**
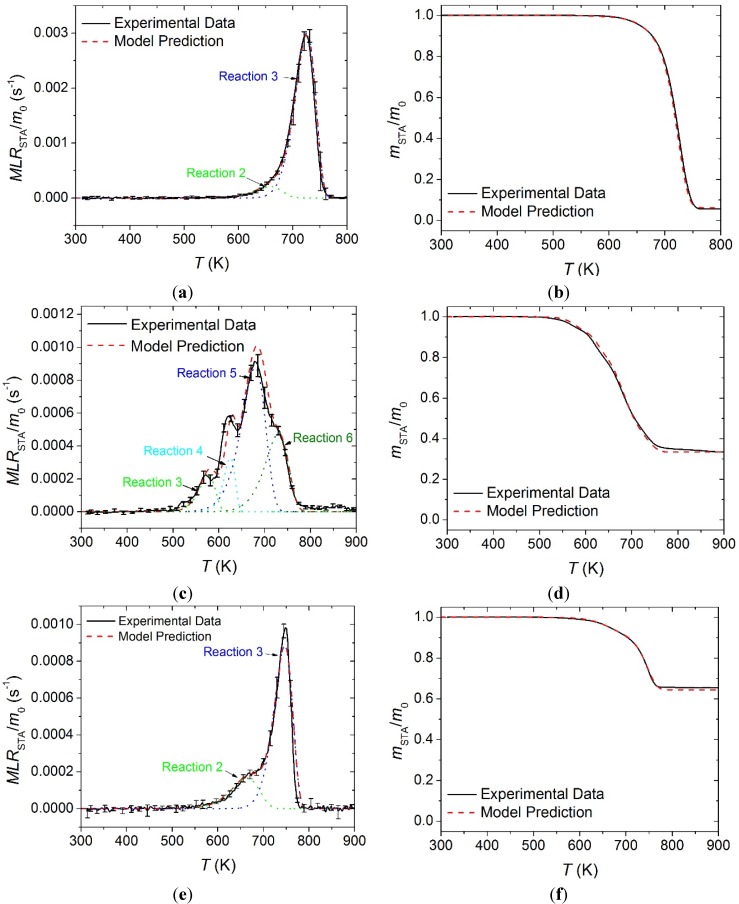
Normalized mass loss rate (*MLR*) and normalized mass data collected in STA experiments and model predicted curves for: (**a**) and (**b**) face yarn layer; (**c**) and (**d**) the middle layer; and (**e**) and (**f**) the base layer. Error bars indicate two standard deviations of the mean experimental data.

The STA normalized mass and normalized *MLR* data for all carpet layers is plotted in Figure 7 along with the curves predicted by the reaction mechanism shown in Table 1. In Figure 7, *m*_0_ indicates initial mass of the sample. In general, the reaction mechanisms determined in this work generated curves that agreed well with the experimental *MLR* and total mass curves. All error bars displayed in this work correspond to two standard deviations of the mean.

The heat flow rate data collected in STA tests on each layer were analyzed to determine the temperature-dependent heat capacity for all the virgin carpet components. Two linear temperature-dependent relationships were found for the Face Yarn_virgin_ and Face Yarn_melt_ components. The heat capacity of all the Middle_virgin_ components was adequately described with a single temperature-dependent term. It was impossible to assign a heat capacity value to each individual Middle_virgin_ component, and all were assigned the same value. The base layer sample melted shortly into the tests, and it proved impossible to determine the heat capacity of the Base_virgin_ component from the collected heat flow rate data. The heat capacity of the Base_melt_ component was determined with a linear temperature-dependence and it was assumed that the same expression could adequately describe the heat capacity of the Base_virgin_ component.

The Face Yarn_char_ and Middle_char_ components were characterized by low masses and a porous structure that compromised the thermal contact between the sample and the crucible and yielded unreliable heat flow rate measurements. These char components were assigned a single heat capacity that was measured as the mean value for the chars produced by seven common polymers [21]. The heat capacity of the Base_char_ component was determined by conducting independent tests on the char produced from thermal degradation of the base layer sample. The heat capacity of the char did not follow a recognizable functional form, so the arithmetic mean of the data over the entire tested temperature range was defined as the heat capacity of the Base_char_ component.

The heat capacity of the Face Yarn_int_. component was defined as the mean between the heat capacities of the Face Yarn_melt_ component evaluated at 560 K and the Face Yarn_char_ component. The heat capacity of the Middle_3,melt_ and Middle_4,melt_ components was defined as the mean between the heat capacity of the Middle_virgin_ components evaluated at 500 K and the Middle_char_ component. The heat capacity of the Base_int_ component was defined as the mean between the heat capacities of the Base_melt_ component evaluated at 600 K and the Base_char_ component. The heat capacity of the reactant was evaluated at a different temperature for each layer based on the temperature at which the intermediate was produced and subsequently reacted.

The heat capacity of the gaseous volatiles produced during thermal degradation of the carpet samples was assumed to be well approximated by hydrocarbons ranging in length from one to eight carbon atoms in the temperature range of 400 K to 500 K. This resulted in the specific heat capacity of all gaseous volatiles defined as 1800 J·kg^−1^·K^−1^. The expressions determined for the heat capacity of each component are provided in Table 2.

**Table 2:** Heat capacity values for each component in the carpet composite.

Component	*c* [J kg^−1^ K^−1^]	Method
Face Yarn_virgin_	8.2*T* − 1180	STA
Face Yarn_melt_	3.6*T* + 580	STA
Face Yarn_int._	2150	Assumed
Face Yarn_char_, Middle_char_	1700	[21]
Middle_1,virgin_, Middle_2,virgin_, Middle_3,virgin_, Middle_4,virgin_	4.2*T*	STA
Middle_3,melt_, Middle_4,melt_	1900	Assumed
Base_virgin_	2.0*T* + 1000	Assumed
Base_melt_	2.0*T* + 1000	STA
Base_int._	1525	Assumed
Base_char_	850	STA
Face Yarn_volatiles_, Middle_volatiles_, Base_volatiles_	1800	Assumed

**Figure 8:**
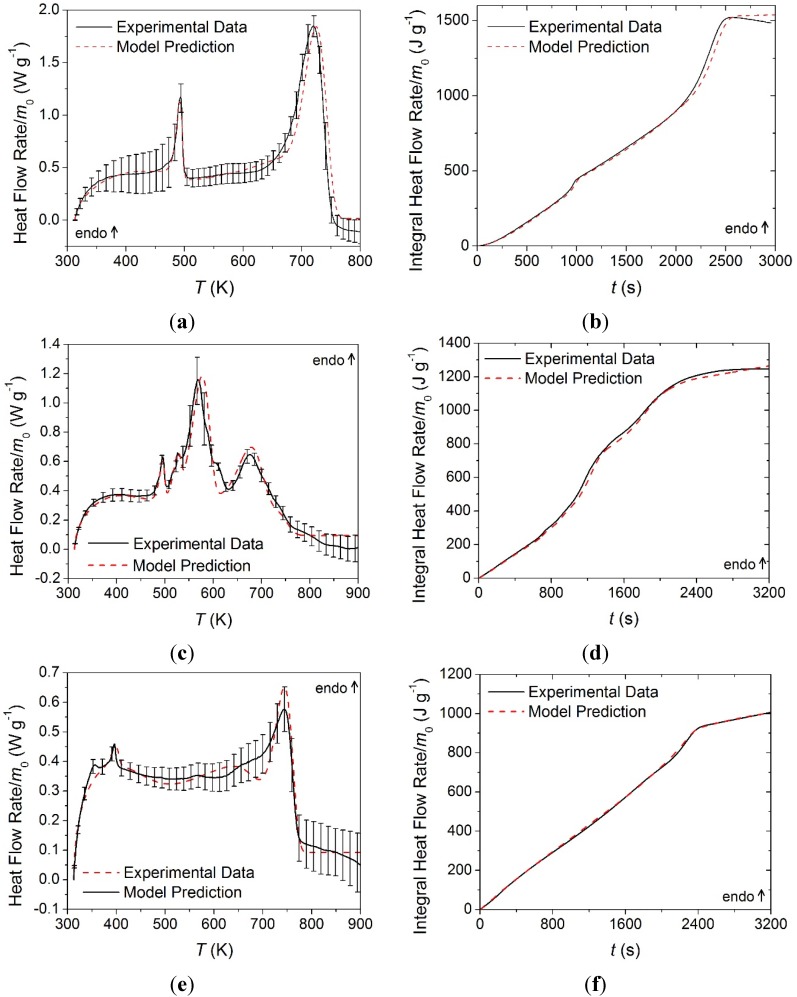
Normalized heat flow rate and integral heat flow rate data collected in STA experiments and model predicted curves for: (**a**) and (**b**) face yarn layer; (**c**) and (**d**) the middle layer; and (**e**) and (**f**) the base layer. Error bars indicate two standard deviations of the mean experimental data.

The integral between the experimental heat flow rate curve and the sensible enthalpy baseline was defined as the enthalpy absorbed by the sample undergoing phase transition or thermal degradation. Each heat of reaction was assigned to the reaction that occurred at the temperature range corresponding to the heat flow rate peak. It was difficult to differentiate between the two thermal degradation reactions in the heat flow rate curve for the face yarn and the base layer and the total integral of the peak was divided between the two reactions in approximate proportion to the total mass volatilized in each reaction. The resulting heat flow quantities associated with each reaction are provided in Table 1. The experimental heat flow rate curves are plotted in Figure 8 along with the model predictions generated with the parameters summarized in Table 1 and Table 2.

#### 4.1.2. Heat of Combustion Determination

**Figure 9:**
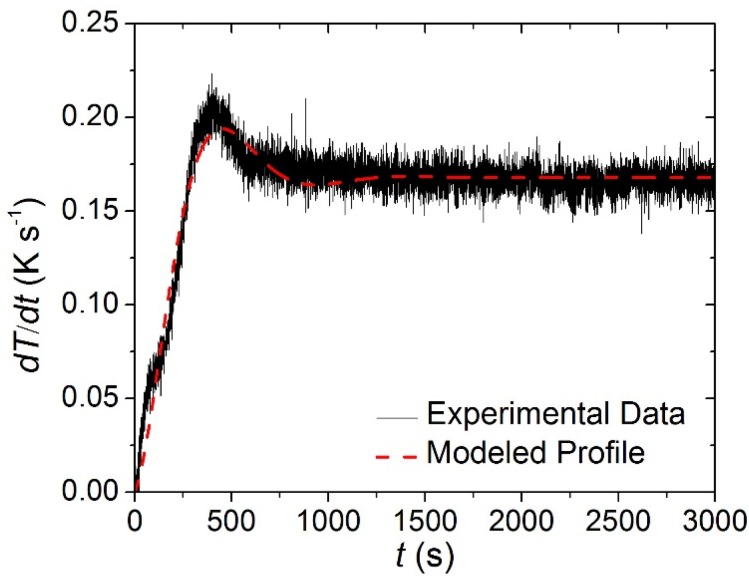
Experimentally observed and modeled heating rate histories typical of the Microscale Combustion Calorimetry (MCC) experiments conducted in this work. The coefficients for Equation (14) that describe the modeled curve are the following: *a* = 0.168 K s^−1^, *b* = 0.0039 s^−1^, *f* = 0.0065 s^−1^, *g* = 0.256.

The MCC data collected on each of the carpet layers at a set heating rate of 0.167 K·s^−1^ were analyzed using the degradation kinetics determined from analysis of STA data. The heating rate profile observed in MCC experiments was different than the profile observed in STA experiments, but was adequately described by the form of Equation (11). The mass loss rate was predicted through a simulation adhering to a heating rate profile that approximated the profile observed in MCC experiments. The agreement between the experimentally observed and modeled heating rate profiles is displayed in Figure 9.

A heat of combustion was assigned to the gaseous components (labeled as volatiles) produced in each of the thermal degradation reactions identified for each material. The mean *HRR* curve measured for each layer was shifted in temperature to properly align with the *MLR* curve predicted with the corresponding reaction mechanism. The magnitude of the shift in the data for each layer was based on the principle that heat release required a concurrent mass loss. By applying this shift, the *MLR* curves agreed well with the rising and falling edges of the *HRR* curves. The face yarn *HRR* curve was shifted upward in temperature by 14 K, the middle layer curve was shifted upward in temperature by 15 K, and the base layer was shifted upward in temperature by 5 K.

**Figure 10:**
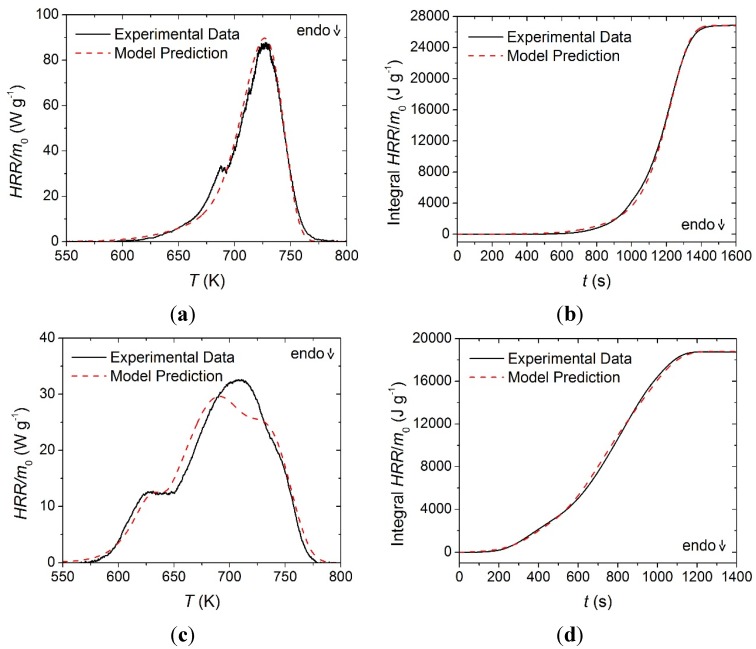

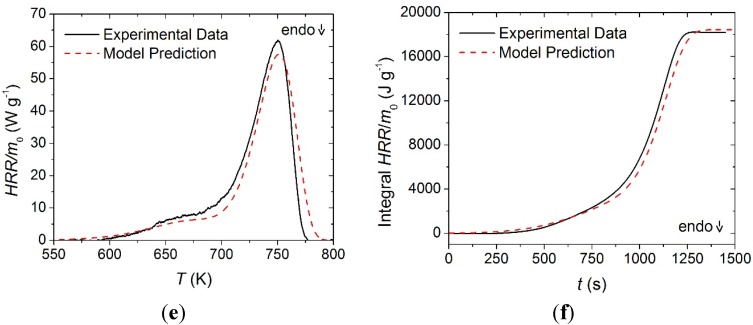
Normalized heat release rate and integral heat release rate data collected in MCC experiments and model predicted curves for: (**a**) and (**b**) the face yarn layer; (**c**) and (**d**) the middle layer; and (**e**) and (**f**) the base layer. Error bars were omitted due to small magnitude scatter.

The offsets between the MCC data and the STA-based kinetics are likely caused by the differences in the heat transport characteristics to and within the crucibles in these two instruments. The current analysis was based on an assumption that the STA provides a more reliable sample temperature control than the MCC. The heats of combustion determined through this analysis are provided in Table 3 and the experimental and predicted *HRR* curves and integral *HRR* curves are provided in Figure 10. The MCC data was highly repeatable for all materials, and error bars were omitted because the magnitude of the scatter was insignificant. Contrary to the convention used to define the heats of reaction, positive values in Table 3 correspond to exothermic processes.

**Table 3:** Effective heat of combustion values for the volatile species released in each reaction. Positive heats of combustion are exothermic.

Volatile Species	*h*_c_ (J kg^−1^)
Face Yarn_volatiles,reaction 2_	2.4 × 10^7^
Face Yarn_volatiles,reaction 3_	2.9 × 10^7^
Middle_volatiles,reaction 3_	0
Middle_volatiles,reaction 4_	1.6 × 10^7^
Middle_volatiles,reaction 5_	2.4 × 10^7^
Middle_volatiles,reaction 6_	5.0 × 10^7^
Base_volatiles,reaction 2_	3.4 × 10^7^
Base_volatiles,reaction 3_	5.9 × 10^7^

The *HRR* curve collected on the middle layer was shifted such that the falling edge of the curve corresponded to the falling edge of the *MLR* curve. After applying this offset in the temperature scale, the first thermal degradation reaction appeared to correspond to a zero magnitude heat release. This led to the conclusion that the volatile species produced in this reaction had no associated heat of combustion. The other values determined for the heats of combustion of volatile species were within a reasonable range for common fuels.

#### 4.1.3. Absorption Coefficient and Emissivity Determination

The reflection loss coefficient, *γ*, accounts for reflection of incident radiation at the interface between the material and the gaseous atmosphere. In this work, this coefficient was assumed to be 0.05 for all undegraded carpet layers. This value is the approximate mean of the reflection loss coefficients for common polymers tabulated by Tsilingiris [26] and supported by the work of Linteris, *et al.* [25] and Försth and Roos [28].

The data collected in transmitted heat flux measurements on all the sample materials and used to calculate the absorption coefficients are provided in Table 4. Since the virgin face yarn was observed to melt shortly after the beginning of radiant heating, the absorption coefficient of the melted face yarn was measured instead of the virgin face yarn. The density of the melted face yarn used in the calculation is discussed later in this section.

**Table 4:** Measurements used to calculate the absorption coefficient for each virgin and melt component.

Layer	$\left(\frac{I_{x=0}}{I_{x=\delta }}\right)$	*δ* (m)	*ρ* (kg m^−3^)	*κ* (m^2^ kg^−1^)
Face Yarn Melt	0.025	0.0008 ± 0.0001	625	7.17
Middle Layer	0.026	0.0013 ± 0.0001	582	4.69
Middle Layer	0.020	0.0016 ± 0.0001	582	4.09
Base Layer	0.010	0.0010 ± 0.0001	1060	4.25
Base Layer	0.005	0.0010 ± 0.0001	1060	4.90

Since the radiant flux transmitted through the melted face yarn was measured, the density used to model the Face Yarn_melt_ component was also used to calculate the absorption coefficient. The densities of the middle layer and the base layer defined in the individual layer models were used to calculate the absorption coefficient of each of those layers. The mean of the individual measurements of the absorption coefficient for each layer was calculated to define the absorption coefficient in the models. Approximate values were assigned to each component based on the transmitted heat flux measurements (7 m^2^·kg^−1^ for the face yarn, 4.4 m^2^·kg^−1^ for the middle layer, and 4.6 m^2^·kg^−1^ for the base layer). The absorption coefficients of the melt and intermediate components were assigned the same absorption coefficient as the virgin component for all layers.

It was observed during gasification tests on the upper layer that the char components were optically dark and appeared to be graphitic. To make the simulations consistent with this observation, the absorption coefficient of the char components was defined such that all radiation was absorbed at the surface of the sample (100 m^2^·kg^−1^). The char formed during thermal degradation of the base layer did not appear to be optically dark. The absorption coefficient for the ${\text{Base}}_{\text{char}}$ component was assumed to be equal to the absorption coefficient of all other base layer components.

The relationship between the reflection loss coefficient and emissivity of optically thick materials (must add to unity) led to the definition of the emissivity of all of the virgin and melt components in each of the carpet layers as 0.95. Due to observations of the samples in gasification tests on each of the layers, the char components were assumed to have lower emissivities than the virgin components. It was assumed that the char components had high carbon content, and the emissivity of all chars was expected to be similar to graphite. The emissivity of graphite was measured at elevated temperatures as approximately 0.86 [29] and was defined as the emissivity of the char components. These definitions are consistent with results of a study by Försth and Roos, who conducted experiments on various colors of PVC and vinyl carpets and found that in some instances, the emissivity tended to decrease as the samples degraded [28]. The emissivity of the intermediate components was defined as the mean value between the virgin components and the char components (ϵ=0.905).

#### 4.1.4. Thermal Conductivity Determination

It was observed that the thickness of the face yarn decreased by a factor of approximately five upon melting. This observation was difficult to confirm in gasification tests because the surface of the sample was completely covered with rapidly regenerating bubbles shortly after melting occurred. However, it was reproduced in a furnace with the temperature set to the melting point of the face yarn (approximately 490 K). A small mass of porous char was produced by the face yarn during degradation, though the thickness of the face yarn layer did not change significantly after melting. The density of the Face Yarn_melt_ component was defined five times larger than the density of the Face Yarn_virgin_ component, and the density of the Face Yarn_int_ and Face Yarn_char_ components were defined proportional to the stoichiometric coefficient for the condensed phase product of each reaction to simulate a constant thickness for the layer after melting.

All Middle_virgin_ components were defined with the same density because it was impossible to identify and separate each individual initial component. Two of the middle layer components underwent phase changes that did not affect the geometry of the sample, so the Middle_melt_ components were defined with the same density as the Middle_virgin_ components. The Base_virgin_ component went through a phase change without the geometry of the layer changing considerably and the density of the ${\text{Base}}_{\text{melt}}$ component was defined equivalent to the virgin component density. The overall thickness of the middle layer and the base layer remained approximately constant throughout the CAPA tests. The densities of the Middle_char_, Base_int_ and Base_char_ components were defined proportional to the associated stoichiometric coefficients in the reaction mechanism to maintain a constant thickness in the simulations. The definitions for the densities of all components are provided in Table 5.

**Table 5:** Full set of thermophysical properties used in the individual upper layer model and base layer model.

Component	*ρ* (kg m^−1^)	*k* (W m^−1^ K^−1^)	ϵ	*κ* (m^2^ κg^−1^)
**Face Yarn**				
Face Yarn_virgin_	125	0.05	0.95	7
Face Yarn_melt_	625	0.05	0.95	7
Face Yarn_int._	575	0.025 + 6.5 × 10^−10^*T*^3^	0.905	7
Face Yarn_char_	34.5	11 × 10^−10^*T*^3^	0.86	100
**Middle Layer**				
Middle_1,virgin_, Middle_2,virgin_, Middle_3,virgin_, Middle_4,virgin_, Middle_3,melt_, Middle_4,melt_	582	0.05	0.95	4.4
Middle_char_	194.4	11 × 10^−10^*T*^3^	0.86	100
**Base Layer**				
Base_virgin_, Base_melt_	1060	0.25 – 2.85 × 10^−4^*T*	0.95	4.6
Base_int._	975.2	0.125 – 1.425 × 10^−4^*T* + 3.5 × 10^−10^*T*^3^	0.905	4.6
Base_char_	692.4	7 × 10^−10^*T*^3^	0.86	4.6

**Figure 11:**
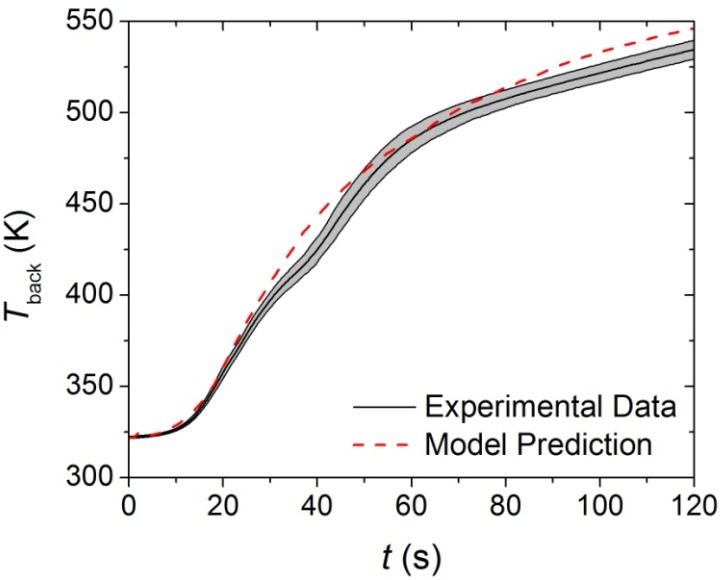
First 120 s of experimental *T*_back_ curve measured in Controlled Atmosphere Pyrolysis Apparatus (CAPA) tests and corresponding model predicted curve for the upper layer exposed to a radiant flux of 30 kW·m^−2^. The shaded region corresponds to two standard deviations of the mean experimental data.

The thermal conductivities of the components in each layer of the carpet composite were the only remaining undefined parameters that affected the pyrolysis model predictions. Inverse analyses were conducted on the *T*_back_ data collected in the CAPA tests using the ThermaKin modeling environment. The initial rise of the *T*_back_ data curve was chosen as the target for the virgin and melt components because these were the only components that affected the *T*_back_ curve early in the tests. The model prediction for the upper layer (face yarn and middle layer) is compared to the experimental data in Figure 11. The temperature prediction was not sensitive to the thermal transport parameters of the char and intermediate components for the time range that corresponded to the initial rise of *T*_back_.

It was found that a single, constant value for the thermal conductivity (*k* = 0.05 W·m^−1^·K^−1^) of the Face Yarn_virgin_, Middle_virgin_, Face Yarn_melt_ and Middle_melt_ components was adequate to describe the rising edge of the *T*_back_ curve. There was no evidence in the data of a change in the thermal conductivity from the virgin components to the melt components. Though this thermal conductivity value is low for a mixture of solid polymers and is more typical of air at elevated temperatures (650 K), the structure of the carpet upper layer supports a thermal conductivity value lower than the typical range for polymers. The face yarn was made of fibrous filaments woven into a yarn and the majority of the volume of the defined face yarn layer was air (or, in the case of the gasification tests, nitrogen). Furthermore, the face yarn melted shortly after exposure to the cone heater and the melted face yarn was characterized by rapidly regenerating bubbles. The middle layer was structured as a mesh interwoven with face yarn and although the density of the middle layer was larger than the face yarn, gases still made a large contribution to the volume of the layer.

Although the thermal conductivity determined for the upper layer is the only value that provides an adequate agreement between the predicted curve and the experimental data, it is probable that some physical phenomena that occurred during testing are not represented in the model. As mentioned in Section 3.2.4, the edges of the upper layer sample tended to curl inward during CAPA tests, and the method of securing the sample to the holder was only partially effective in reducing the deformation. It is possible that the decrease in exposed area may have caused the samples to deviate from one-dimensional behavior. The back surface of the upper layer samples was textured which may have compromised the thermal contact between the sample and the aluminum foil, resulting in inaccurate *T*_back_ profiles.

**Figure 12:**
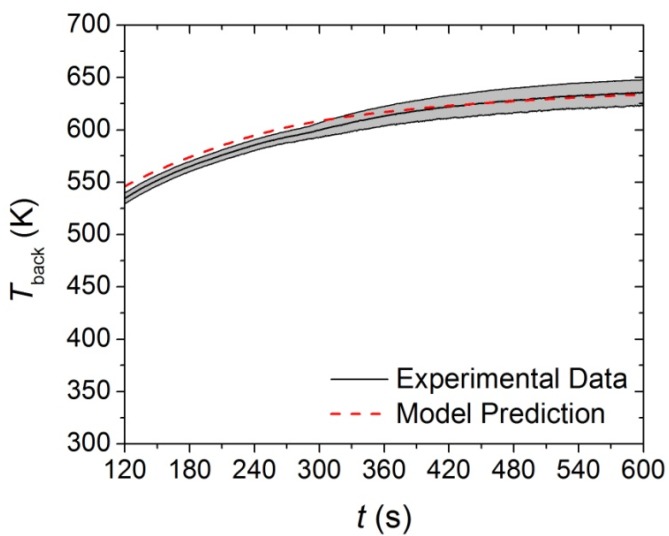
Final 480 s of experimental *T*_back_ curve measured in CAPA tests and corresponding model predicted curve for the upper layer exposed to a radiant flux of 30 kW·m^−2^. The shaded region corresponds to two standard deviations of the mean experimental data.

An inverse analysis was conducted on the *T*_back_ data collected on the upper layer subjected to a heat flux of 30 kW·m^−2^ to determine the thermal conductivities of the char components. The target data for the inverse analysis was chosen as the slowly rising portion of *T*_back_ that was observed after 120 s in the gasification tests. Due to the high porosity of the chars produced in each layer, radiation was assumed to be the dominant mode of heat transfer through the char. The radiation diffusion approximation [30] was invoked to describe the thermal conductivity of all the char components. An attempt was made to describe all the char components with a single thermal conductivity expression of the form *βT*^3^ and it was determined that a single value of *β* adequately described the *T*_back_ profile in the final 480 s of the curve. The thermal conductivities of all the intermediate components were defined as the mean of the thermal conductivities of the corresponding virgin component and char component. For the face yarn intermediate, this produced an expression with a constant term and a *T*^3^ term. The agreement between the *T*_back_ predictions and the experimental data collected on the upper layer at a heat flux of 30 kW·m^−2^ are shown in Figure 12. The full set of parameters that were determined for the upper layer of the carpet composite to describe thermal transport to and within the solid sample are provided in Table 5.

**Figure 13:**
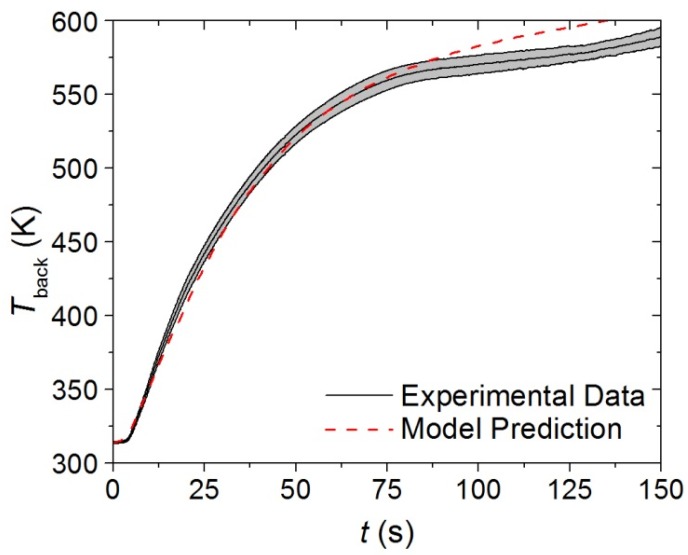
First 150 s of experimental *T*_back_ curve measured in CAPA tests and corresponding model predicted curve for the base layer exposed to a radiant flux of 30 kW·m^−2^. The shaded region corresponds to two standard deviations of the mean experimental data.

An inverse analysis to determine the thermal conductivity of the virgin and melt components was conducted on the data collected in CAPA tests on the base layer at a heat flux of 30 kW·m^−2^. The target data was identified as the slope of the initial increase in the *T*_back_. Inadequate agreement between the model prediction and the experimental data was produced with a single, constant value for the thermal conductivity of the virgin and melt components. A linearly decreasing thermal conductivity for the ${\text{Base}}_{\text{virgin}}$ and ${\text{Base}}_{\text{melt}}$ components was able to adequately predict both the fast and slow rising portions of the initial 150 s of the *T*_back_ curve. The agreement between the experimental curve and the model prediction are provided in Figure 13.

**Figure 14:**
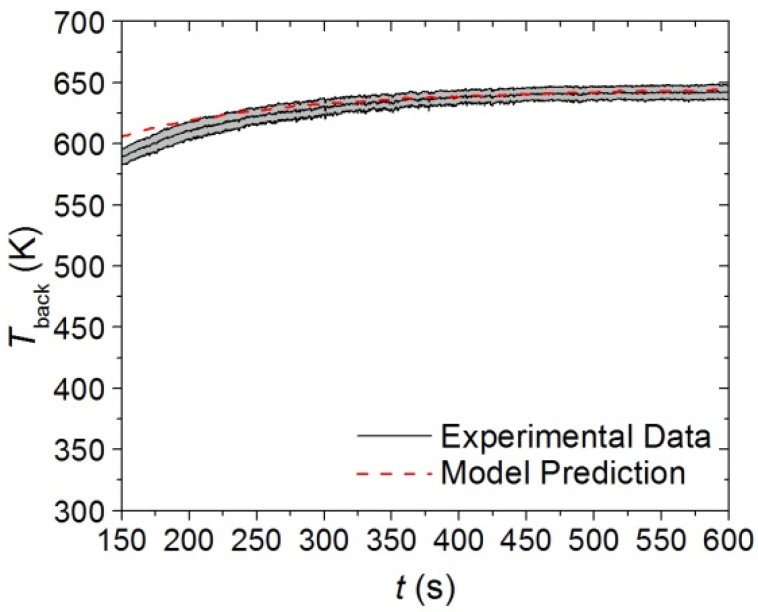
Final 450 s of experimental *T*_back_ curve measured in CAPA tests and corresponding model predicted curve for the base layer exposed to a radiant flux of 30 kW·m^−2^. The shaded region corresponds to two standard deviations of the mean experimental data.

The data collected in the CAPA tests on the base layer were analyzed to determine the thermal conductivity of the ${\text{Base}}_{\text{char}}$ component. The resulting experimental and predicted curves for the *T*_back_ of the base layer are provided in Figure 14. A single, constant value of *β* in the *βT*^3^ expression adequately described the *T*_back_ profile in the final 450 s of the curve. The thermal conductivity of the Base_int_ component was defined as the mean between the Base_virgin_ and Base_char_ components, which resulted in a form with a constant, linear, and *T*^3^ term. The full set of parameters that define the base layer thermophysical properties are provided in Table 5.

#### 4.1.5. Parameter Uncertainty

The estimated uncertainties in the pre-exponential factors were ±50%, in the activation energy were ±3%, and in the stoichiometric coefficients were ±5%. The density definitions of all components was assumed to be ±10% on average, based on the discussion of the geometric and gravimetric measurements made on the composite material and layers. The heats of reaction and heats of combustion each had an estimated uncertainty of approximately ±20% due to the graphical method by which they were determined. Though the individual heats of reaction and heats of combustion were determined with relatively high uncertainty, it was shown in previous sections that the total heat released during combustion and heat absorbed throughout degradation are within ±5%. The heat capacity values determined for the virgin and melt components carried an uncertainty of approximately ±10% due to relatively low magnitude scatter in the data. The heat capacity measured for the base char had an uncertainty of approximately ±20% due to more significant scatter in the experimental data. The uncertainty in the values of the thermal conductivity defined for all components was approximated as ±15% based on changes in the agreement between the model predictions and the experimental curves with changes to the thermal conductivity definitions. Though Linteris estimated the uncertainty in the measurement of the absorption coefficients as ±5%, the variation in the values measured here indicate that an uncertainty of approximately ±25% is more representative.

### 4.2. Individual Layer Model Predictions

#### 4.2.1. Upper Layer (Consisting of Face Yarn and Middle Layer)

**Figure 15:**
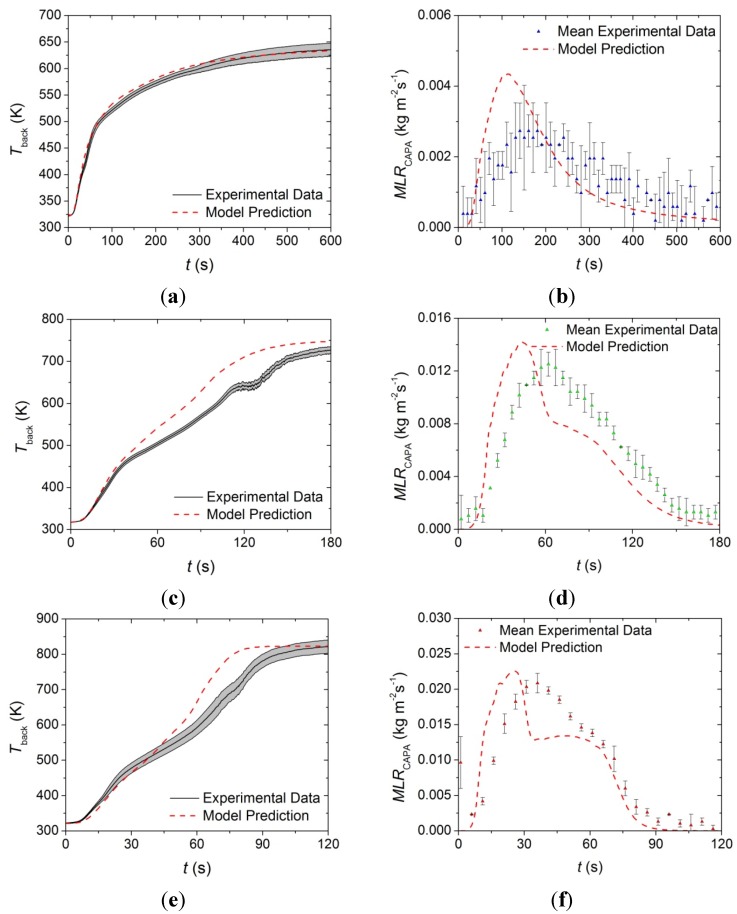
Experimental *T*_back_ and *MLR* curve collected in CAPA tests and corresponding model predicted curve for the upper layer exposed to radiant fluxes of (**a**) and (**b**) 30 kW·m^−2^; (**c**) and (**d**) 50 kW·m^−2^; and (**e**) and (**f**) 70 kW·m^−2^. The shaded region and error bars correspond to two standard deviations of the mean experimental data.

The model constructed from the parameters presented in Table 1, Table 2 and Table 5 was independently validated against *T*_back_ data collected at incident heat fluxes of 50 and 70 kW·m^−2^ as well as *MLR* curves collected in CAPA tests conducted at incident heat fluxes of 30, 50, and 70 kW·m^−2^. The model predicted curves and the experimental data are provided in Figure 15.

The model for the upper layer captures the time to the initial increase of the *T*_back_ profile as well as the slope of the initial increase for all incident heat fluxes. The model also accurately predicts the final steady temperature at each heat flux. The model overpredicts the *T*_back_ from approximately 40 to 180 s at a heat flux of 50 kW·m^−2^ and overpredicts the *T*_back_ from 45 to 90 s at a heat flux of 70 kW·m^−2^, although the qualitative shape of each temperature prediction agrees with the experimental data. This overprediction may be attributed to systematic errors in the measurement of *T*_back_ due to sample deformation of the upper layer and poor thermal contact between the back surface of the sample and the aluminum foil. It may also be due to uncertainty in the measurement because of degradation of the high emissivity paint on the back surface of the foil above 600 K.

The model was able to accurately predict the total mass lost at all heat fluxes and the qualitative shapes of each *MLR* curve. The approximate time to initial mass loss and the approximate peak *MLR* were also well predicted at each heat flux. The model systematically overpredicted the rising edge of the *MLR* curve and underpredicted the time to the peak *MLR*. There was also a portion of the predicted curve after the peak *MLR* that underpredicted the experimental data at all heat fluxes.

The disagreement between the predicted *MLR* curves and the experimental curves may be attributed to possible compensation effects between the absorption coefficients and the thermal conductivities for the face yarn components. The measurements made to determine the absorption coefficient of the face yarn were conducted on a melted sample that had solidified, but the melted face yarn observed in the tests was characterized by rapidly regenerating bubbles that may have influenced the actual absorption coefficient and thermal conductivity of the layer. Though there was a possible compensation effect, and considering the complexity of the layer, the predictions provide adequate agreement with the experimental data over a range of conditions outside of the calibration conditions.

#### 4.2.2. Base Layer

**Figure 16:**
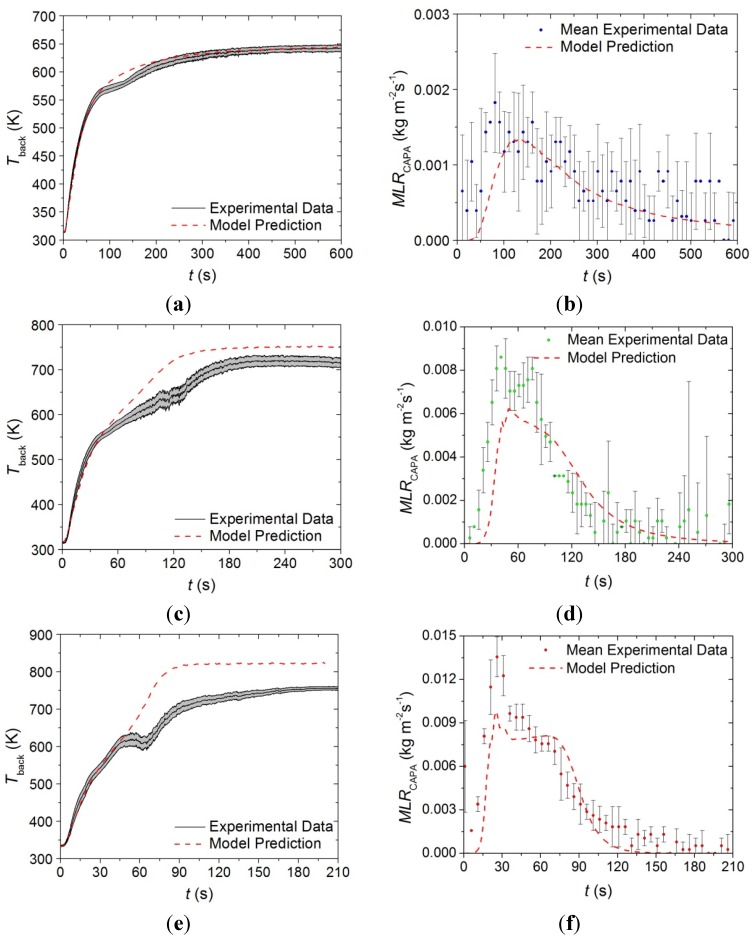
Experimental *T*_back_ and *MLR* curves collected in CAPA tests and corresponding model predicted curves for the base layer exposed to radiant fluxes of (**a**) and (**b**) 30 kW·m^−2^, (**c**) and (**d**) 50 kW·m^−2^, and (**e**) and (**f**) 70 kW·m^−2^. The shaded region and error bars correspond to two standard deviations of the mean experimental data.

The model constructed from the parameters for the base layer presented in Table 1, Table 2 and Table 5 was independently validated against *T*_back_ data collected at incident heat fluxes of 50 and 70 kW·m^−2^ as well as *MLR* curves collected at incident heat fluxes of 30, 50, and 70 kW·m^−2^. The model predicted curves and the experimental data are provided in Figure 16.

The model tends to predict the experimentally observed time to the initial temperature rise and the slope of the initial temperature rise well at all incident heat fluxes. The model tends to overpredict *T*_back_ after about 50 s at heat fluxes of 50 and 70 kW·m^−2^, although the temperatures measured at times later than 50 s into the tests for the higher heat fluxes correspond to temperatures significantly above 600 K, so there is some uncertainty about the validity of that data due to degradation of the high emissivity paint on the back surface of the sample. It is also possible that the glass reinforcement, which comprises a large fraction of the residual mass in the base layer and has a relatively low emissivity, compromised the well-defined emissivity at the back surface. A decrease in the emissivity of the measured surface manifests as artificially low *T*_back_ measurements.

The predicted *MLR* profiles tend to capture the overall trends in the experimental data at all heat fluxes. The initial rise to the peak *MLR* for the predicted curves follows the slope of each of the experimental curves with a slight lag in the time to the initial increase. The peak value is slightly underpredicted in each case, although the time to the peak *MLR* is captured well by the model.

### 4.3. Full Carpet Model Predictions

The individual upper layer and base layer model parameters that were validated against *T*_back_ and *MLR* data from CAPA tests were combined in a full carpet model to evaluate their ability to predict the pyrolysis behavior of the full carpet composite. The thicknesses and densities of the middle layer and the base layer were modified based on a discrepancy in thickness measurements discussed in Section 3.1. The density definitions for the full carpet composite model are provided in Table 6.

**Table 6:** Thermal conductivity and density values for Final Full Carpet model. Modifications to property values from individual layer models are shown in bold.

Component	*ρ* (kg m^−1^)	*k* (W m^−1^ K^−1^)
**Face Yarn**		
Face Yarn_virgin_	125	**0.12**
Face Yarn_melt_	625	**0.12**
Face Yarn_int._	575	**0.06 + 3.5 × 10^−10^*T*^3^**
Face Yarn_char_	34.5	**7 × 10^−10^*T*^3^**
**Middle Layer**		
Middle_1,virgin_, Middle_2,virgin_, Middle_3,virgin_, Middle_4,virgin_, Middle_3,melt_, Middle_4,melt_	**750**	**0.12**
Middle_char_	**250.5**	**7 × 10^−10^*T*^3^**
**Base Layer**		
Base_virgin_, Base_melt_	**1200**	0.25 – 2.85 × 10^−4^*T*
Base_int._	**1104**	0.125 – 1.425 × 10^−4^*T* + 3.5 × 10^−10^*T*^3^
Base_char_	**783.8**	7 × 10^−10^*T*^3^

**Figure 17:**
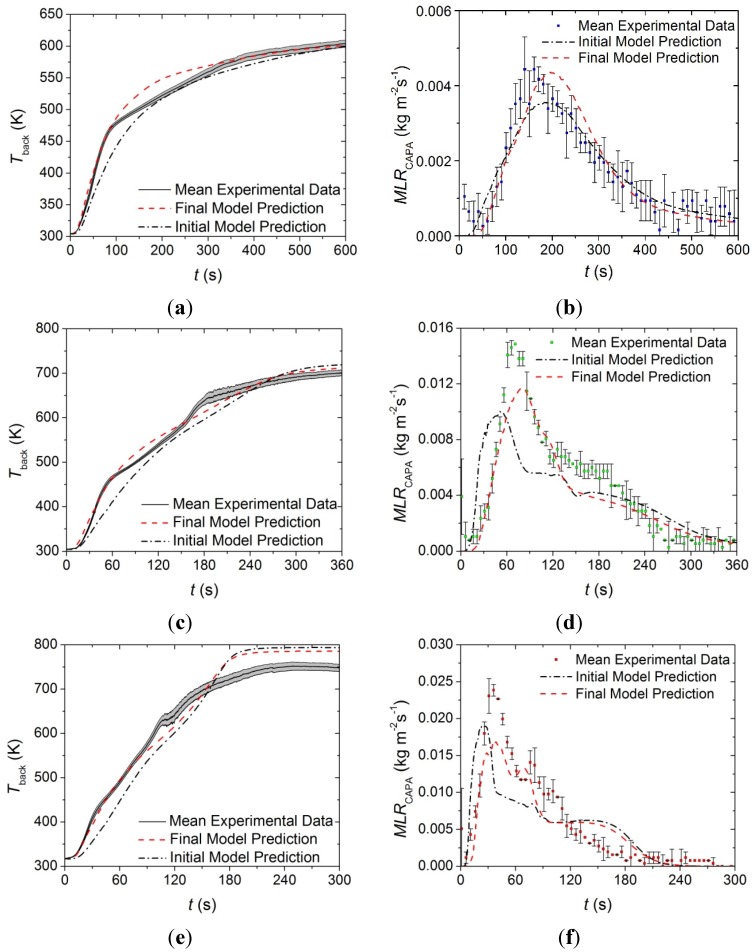
Experimental *T*_back_ and *MLR* curve collected in CAPA tests and corresponding model predicted curves for the full carpet composite exposed to radiant fluxes of (**a**) and (**b**) 30 kW·m^−2^, (**c**) and (**d**) 50 kW·m^−2^, and (**e**) and (**f**) 70 kW·m^−2^. The shaded region and error bars correspond to two standard deviations of the mean experimental data.

The *T*_back_ and *MLR* data predicted by the model of the full carpet constructed from the combination of the individually parameterized upper and base layer representations are labeled in Figure 17 as “Initial Model Prediction”. The model was able to predict the qualitative trends in the experimental *T*_back_ curves at all heat fluxes. The approximate steady *T*_back_ was well predicted at 30 and 50 kW·m^−2^ and slightly overpredicted at 70 kW·m^−2^. The shape of the *MLR* curve was well predicted at 30 kW·m^−2^, but the agreement between the predicted curve and the experimental curve degraded at the higher heat fluxes. Though the model was able to predict the qualitative trends in the experimental data, the quantitative agreement required improvement.

All predicted temperature and *MLR* curves had a tendency to be lower than the experimental curves. This tendency may be attributed to the difficulties encountered with testing and modeling the upper layer that led to a low thermal conductivity defined for the virgin face yarn and virgin middle layer components. It is also evident that, by separating the layers, the physical structure of the composite was compromised and the thermal transport within the sample was affected. To investigate the extent to which the structure of the carpet and interaction between layers affects thermal transport in the composite, the upper layer thermal transport was re-parameterized in the context of the full carpet composite.

The full carpet samples did not deform significantly during tests and the texture of the back surface of the full carpet samples guaranteed proper thermal contact between the sample and the aluminum foil. The target data for the inverse analysis to re-parameterize the upper layer was the *T*_back_ profile collected on the full carpet samples in CAPA tests conducted at a heat flux of 30 kW·m^−2^. It was hypothesized that the individually parameterized base layer model provided sufficient description of the actual tested base layer and the only independent parameters that were adjusted to improve agreement between the experimental data and the model prediction were the thermal conductivities of the upper layer components. The curves that were predicted when the thermal transport parameters were adequate to describe the target experimental data are plotted as the “Final Model Prediction” in Figure 17. The changes made to the thermal conductivity definitions provided in Table 5 to generate the “Final Model Prediction” are provided in Table 6.

There is an obvious improvement in the model predictions from the combination of the individually parameterized layers to the re-parameterization of the upper layer in the context of the full carpet composite. The temperature profiles predicted with the final full composite model captured the time to the initial increase in the *T*_back_ and the slope of the initial increase at all heat fluxes. The entire *T*_back_ curve was well predicted at heat fluxes of 30 and 50 kW·m^−2^, and alternated between underpredicting and overpredicting the *T*_back_ above 600 K at a heat flux of 70 kW·m^−2^. An interesting observation is that the experimental *T*_back_ of the base layer and the full composite never reached temperatures higher than approximately 750 K, which corresponds to the peak *MLR* in the base layer TGA data. This temperature indicates the point in the tests at which the largest fraction of the volatile mass of the base layer is liberated from the solid, which leaves a matrix of fiberglass as residue. It is possible that a more comprehensive definition of the base layer that includes the properties of the fiberglass reinforcement would improve the agreement between the experimental data and the model predictions for the full carpet composite.

The initial increase in the *MLR* curve was predicted well by the model at all heat fluxes. The peak *MLR* was well predicted at 30 kW·m^−2^, but the time to the peak was over predicted by approximately 50 s. At the higher heat fluxes, the time to the peak *MLR* was better predicted, but the peak value was underpredicted by larger percentages with each increase in the incident heat flux. The qualitative shape of the predicted curve at 30 and 50 kW·m^−2^ agree with the experimental curve. The curve predicted at a heat flux of 70 kW·m^−2^ showed a slowly decaying plateau from approximately 120 to 210 s that did not occur in the experiments. The mean error between the predicted *MLR* and the mean experimental *MLR* was calculated as 13% for 30 kW·m^−2^ (mean *MLR* value of 0.00171 kg m^−2^·s^−1^), 18% for 50 kW·m^−2^ (mean *MLR* value of 0.00427 kg·m^−2^·s^−1^), and 28% for 70 kW·m^−2^ (mean *MLR* value of 0.00850 kg·m^−2^·s^−1^). The mean error between the predicted *MLR* and the mean experimental *MLR* was within the mean experimental uncertainty for all tested heat fluxes.

## 5. Conclusions

This work presents several improvements to a relatively new methodology to parameterize pyrolysis models [12] and extends this methodology to a multilayered composite system. To the knowledge of the authors, this is the most complicated material system ever to be fully parameterized for a pyrolysis model. The developments to the existing methodology presented here include a focus on characterizing each layer of the composite individually, modeling STA data according to an approximation of the observed heating rate profiles, and the use of MCC tests to incorporate heat release rate predictions into the capabilities of the pyrolysis models.

The carpet sample was divided into three separate layers and the thermal degradation of each was characterized independently. The kinetics and energetics of the thermal degradation process were determined through an inverse analysis of STA data. The heats of combustion of the gaseous species produced in each degradation reaction were determined through analysis of data collected in MCC tests. The absorption coefficient of each layer of the initial material was calculated from data on the radiant flux transmitted through thin film samples. The carpet was divided into two layers for bench-scale gasification tests. These layers were independently investigated to characterize the thermal transport through the carpet composite by conducting inverse analyses on the back surface temperature data collected in these tests.

The models for the two individual layers of the carpet that were tested in the gasification experiments generated *MLR* and *T*_back_ predictions that agreed well with the experimental data. The combination of these two layers produced predictions that had a fair agreement with experimental data collected on the full carpet composite. It was likely that, by separating the layers of the carpet and effectively compromising the structure of the composite, the thermal transport characteristics of the layers were affected. Qualitatively and quantitatively improved predictions were produced by re-parameterizing the thermal conductivity of the upper layer components in the context of the full carpet composite.

The previously used parametrization methodology enhanced by the aforementioned improvements produces pyrolysis models that are capable of predicting the fire response for highly complicated materials subjected to a wide range of conditions. It was shown that individual layer parameterization works to a significant degree and provides the ability to extrapolate results to different material structures, provided the properties of the additional material elements are available. It was also shown that the interfaces between macrostructural elements affect the overall heat transfer within the condensed phase, and high fidelity models require additional measurements on the structures that include any such interfaces for accurate characterization.

## Nomenclature

**Symbols**	**Description**
*A*	Arrhenius pre-exponential factor ((m^3^ kg^−1^)^n−1^s^−1^) (for reaction of order n)
*E*	activation energy (J mol^−1^)
*I*	radiant flux (W m^−2^)
*J*	mass flux (kg m^−2^ s^−1^)
*R*	universal gas constant (J mol^−1^ K^−1^)
*T*	temperature (K)
*c*	heat capacity (J kg^−1^ K^−1^)
*h*	heat evolved in reaction (J kg^−1^)
*k*	thermal conductivity (W m^−1^ K^−1^)
*m*	mass (kg)
*q*	heat flux due to thermal conduction (W m^−2^)
*r*	reaction rate (kg m^−3^ s^−1^)
*t*	time (s)
*x*	Cartesian coordinate (m)
	Greek
*β*	coefficient of cubic term in effective thermal conductivity (W m^−1^ K^−4^)
γ	reflection loss coefficient
*δ*	thickness of sample (m)
ϵ	emissivity
*κ*	absorption coefficient (m^2^ kg^−1^)
*λ*	mass transport coefficient (m^2^ s^−1^)
*ν*	stoichiometric coefficient
*ξ*	mass concentration (kg m^−3^)
*ρ*	density (kg m^−3^)
*σ*	Stefan-Boltzmann constant (W m^−2^ K^−4^)
	Acronyms
CAPA	Controlled Atmosphere Pyrolysis Apparatus
DSC	Differential Scanning Calorimetry
*HRR*	heat release rate
*MLR*	mass loss rate
MCC	Microscale Combustion Calorimeter
STA	Simultaneous Thermal Analysis
TGA	Thermogravimetric Analysis
