# Correction: McKinnon M.B. and Stoliarov S.I. Pyrolysis Model Development for a Multilayer Floor Covering. *Materials* 2015, *8*, 6117–6153

**DOI:** 10.3390/ma8115402

**Published:** 2015-11-11

**Authors:** Mark B. McKinnon, Stanislav I. Stoliarov

**Affiliations:** Department of Fire Protection Engineering, 3106 J.M. Patterson Building, University of Maryland, College Park, MD 20742, USA; mckinn@umd.edu

The authors wish to make the following corrections to this manuscript [1]. During the publishing process, symbols that represented the absorption coefficient in Table 4 and thermal conductivity in Table 5 were changed such that they were inconsistent with the rest of the manuscript. Also, several of the entries in Table 6 were not presented with a bold typeface, although they should have been, as described in the caption of the table. The tables with the correct symbols and formatting are shown below. While the authors are not responsible for these errors, they regret any inconvenience or misunderstanding caused by them.

**Table 4:** Measurements used to calculate the absorption coefficient for each virgin and melt component.

Layer	$\left(\frac{I_{x=0}}{I_{x=\delta }}\right)$	*δ* (m)	*ρ* (kg·m^−3^)	*κ* (m^2^·kg^−1^)
Face Yarn Melt	0.025	0.0008 ± 0.0001	625	7.17
Middle Layer	0.026	0.0013 ± 0.0001	582	4.69
Middle Layer	0.020	0.0016 ± 0.0001	582	4.09
Base Layer	0.010	0.0010 ± 0.0001	1060	4.25
Base Layer	0.005	0.0010 ± 0.0001	1060	4.90

**Table 5:** Full set of thermophysical properties used in the individual upper layer model and base layer model.

Component	*ρ* (kg·m^−3^)	*k* (W·m^−1^·K^−1^)	ϵ	*κ* (m^2^·kg^−1^)
**Face Yarn**				
Face Yarn_virgin_	125	0.05	0.95	7
Face Yarn_melt_	625	0.05	0.95	7
Face Yarn_int._	575	0.025 + 6.5 × 10^−10^*T*^3^	0.905	7
Face Yarn_char_	34.5	11 × 10^−10^*T*^3^	0.86	100
**Middle Layer**				
Middle_1,virgin_, Middle_2,virgin_, Middle_3,virgin_, Middle_4,virgin_, Middle_3,melt_, Middle_4,melt_	582	0.05	0.95	4.4
Middle_char_	194.4	11 × 10^−10^*T*^3^	0.86	100
**Base Layer**				
Base_virgin,_ Base_melt_	1060	0.25 – 2.85 × 10^−4^*T*	0.95	4.6
Base_int._	975.2	0.125 – 1.425 × 10^−4^*T* + 3.5 × 10^−10^*T*^3^	0.905	4.6
Base_char_	692.4	7 × 10^−10^*T*^3^	0.86	4.6

**Table 6:** Thermal conductivity and density values for Final Full Carpet model. Modifications to property values from individual layer models are shown in bold.

Component	*ρ* (kg·m^−3^)	*k* (W·m^−1^·K^−1^)
**Face Yarn**		
Face Yarn_virgin_	125	**0.12**
Face Yarn_melt_	625	**0.12**
Face Yarn_int._	575	**0.06 + 3.5 × 10^−10^*T*^3^**
Face Yarn_char_	34.5	**7 × 10^−10^*T*^3^**
**Middle Layer**		
Middle_1,virgin_, Middle_2,virgin_, Middle_3,virgin_, Middle_4,virgin_, Middle_3,melt_, Middle_4,melt_	**750**	**0.12**
Middle_char_	**250.5**	**7 × 10^−10^*T*^3^**
**Base Layer**		
Base_virgin,_ Base_melt_	**1200**	0.25 – 2.85 × 10^−4^*T*
Base_int._	**1104**	0.125 – 1.425 × 10^−4^*T* + 3.5 × 10^−10^*T*^3^
Base_char_	**783.8**	7 × 10^−10^*T*^3^
